# Management of portal hypertension and ascites in polycystic liver disease

**DOI:** 10.1111/liv.14245

**Published:** 2019-09-20

**Authors:** Lucas H. P. Bernts, Joost P. H. Drenth, Eric T. T. L. Tjwa

**Affiliations:** ^1^ Department of Gastroenterology and Hepatology Radboud Institute for Molecular Life Sciences Radboud University Medical Center Nijmegen The Netherlands

**Keywords:** ascites, Budd‐Chiari syndrome, caval vein, diuretics, hepatic veins, hepatic venous outflow obstruction, polycystic liver disease, portal hypertension, portal vein, somatostatin analogues, stents, surgery

## Abstract

Patients suffering from polycystic liver disease may develop Hepatic Venous Outflow Obstruction, Portal Vein Obstruction and/or Inferior Caval Vein Syndrome because of cystic mass effect. This can cause portal hypertension, leading to ascites, variceal haemorrhage or splenomegaly. For this review, we evaluate the evidence to provide clinical guidance for physicians faced with this complication. Diagnosis is made with imaging such as ultrasound, computed tomography or magnetic resonance imaging. Therapy includes conventional therapy with diuretics and paracentesis, and medical therapy using somatostatin analogues. Based on disease phenotype various (non‐)surgical liver‐volume reducing therapies, hepatic or portal venous stenting, transjugular intrahepatic portosystemic shunts and liver transplantation may be considered. Because of complicated anatomy, use of high‐risk interventions and lack of empirical evidence, patients should be treated in expert centres.

**Abbreviations**
ADPKD: autosomal dominant polycystic kidney disease
ADPLD: autosomal dominant polycystic liver disease
ARPKD: autosomal recessive polycystic kidney disease
BCS: Budd‐Chiari syndrome
CT: computed tomography
hTLV: height‐adjusted liver volume
HVOO: hepatic venous outflow obstruction
HVPG: hepatic vein pressure gradient
ICV: inferior caval vein
ICVS: inferior caval vein syndrome
MRI: magnetic resonance imaging
PLD: polycystic liver disease
PVO: portal vein obstruction
RAAS: Renin‐Angiotensin‐Aldosteron‐System
SSA: somatostatin analogues
TAE: transcatheter arterial embolization
TIPS: transjugular intrahepatic portosystemic shunts
UDCA: ursodeoxycholic acid
US: ultrasound
V2: vasopressin 2

**Key points**

- Portal hypertension is a rare but severe complication of polycystic liver disease
- Patients may suffer from obstruction of hepatic, portal or caval veins
- Diagnostics should focus on imaging techniques
- Treatment should be tailored to each individual patient's symptoms
- Interventional treatment should be performed in specialist centres

## 1 INTRODUCTION

Polycystic liver disease (PLD) is characterized by the presence of numerous fluid‐filled cysts in the liver. PLD occurs in two distinct genetic disorders, associated with autosomal dominant polycystic kidney disease (ADPKD) or in absence of renal cysts as autosomal dominant polycystic liver disease (ADPLD).^1^ PLD is a hereditary condition that results in progressive hepatomegaly in a proportion of patients with subsequent displacement of adjacent organs and symptoms such as pain, dyspnoea, early satiety, hepatic cyst infections and the development of portal hypertension.^1^, ^2^ Disease severity is classified as mild when height‐adjusted liver volume (hTLV) is below 1600 mL/m, moderate between 1600 and 3200 mL/m, and severe above 3200 mL/m.^3^ PLD may result in clinically significant portal hypertension through various mechanisms with variable treatment options. However, literature is scarce on when and how these options come into play. We aim to review the literature on portal hypertension in PLD and discuss management of related complications. Portal hypertension in context of autosomal recessive polycystic kidney disease with congenital hepatic fibrosis is not within the scope of this review.^4^

### 1.1 Causes of portal hypertension in PLD

**Figure 1:**
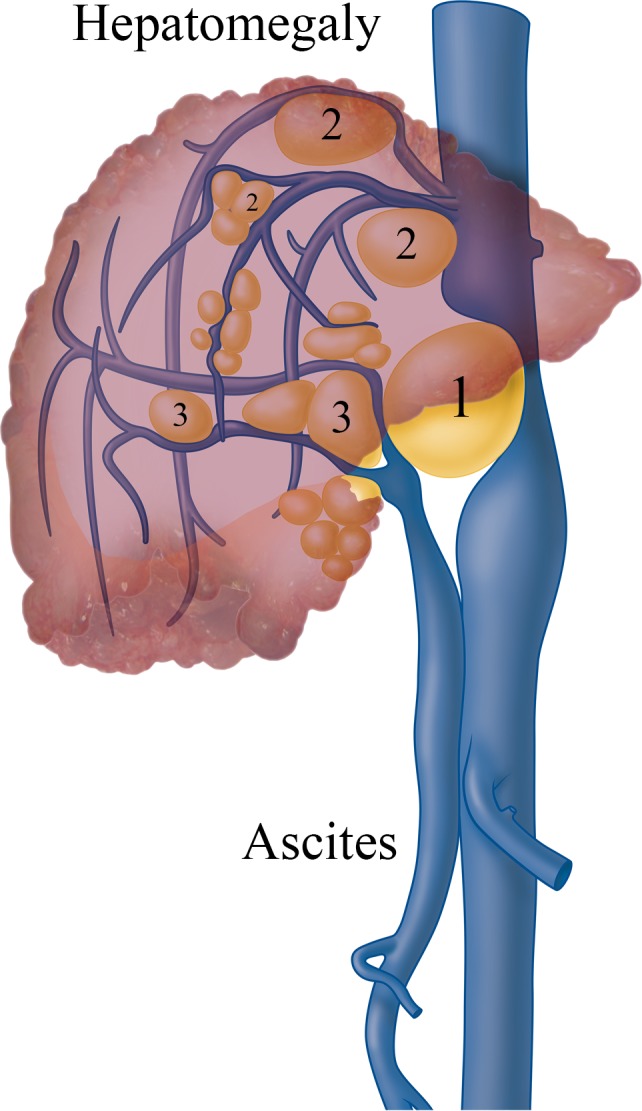
Causes of portal hypertension in polycystic liver disease are shown: 1. inferior caval vein syndrome (ICVS); 2. hepatic venous outflow obstruction (HVOO); 3. portal vein obstruction (PVO)

Clinically significant portal hypertension is a clinical syndrome characterized by splenomegaly, ascites, gastrointestinal varices and encephalopathy and is defined by an increased hepatic venous pressure gradient (HVPG).^5^ Although portal hypertension is mostly associated with cirrhosis, it can also occur in advanced cases of PLD. Patients may be confronted with three typical types of vascular obstruction: (a) Hepatic Venous Outflow Obstruction (HVOO), (b) portal vein obstruction (PVO) and/or (c) inferior caval vein syndrome (ICVS) because of cystic mass effect or unfavorably located cysts. These conditions can lead to non‐cirrhotic portal hypertension.^6^ An anatomical representation of the three vascular obstruction types is presented in Figure 1.

**Figure 2:**
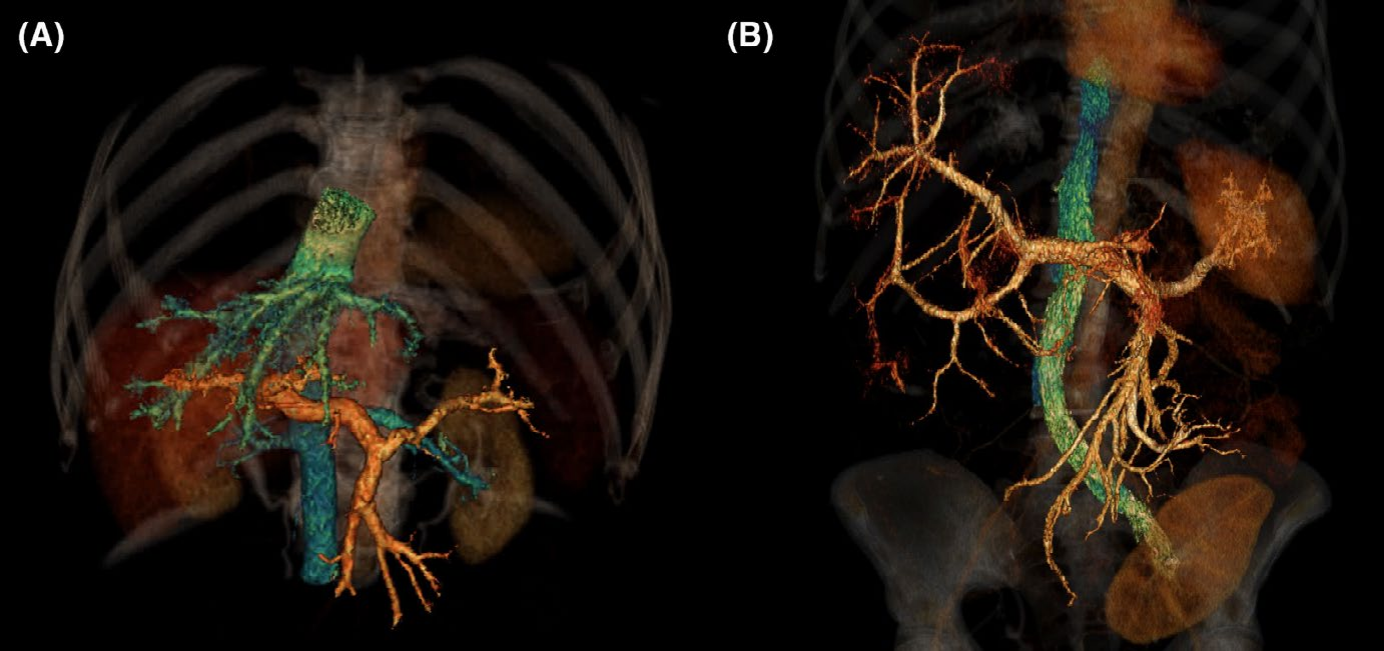
3D‐reconstruction of intravenous contrast, the portal venous system is coloured orange and the caval venous system is coloured cyan. (Panel A) healthy control. (Panel B) patient with polycystic liver disease and hepatic venous outflow obstruction. No hepatic veins are visible due to external compression by cystic liver tissue. Renal transplant is also visible

Hepatic venous outflow obstruction is characterized by reduction in the outflow of venous blood from the liver into the caval vein (Figure 2). HVOO is a rare condition, which can either be caused by (I) hepatic vein thrombosis in Budd‐Chiari Syndrome (BCS), (II) external compression by tumour, cyst or abscess, or (III) after liver transplantation.^7^, ^8^

Portal vein obstruction is characterized by obstruction of inflow from the portal venous system into the liver. This may be because of external compression, but also by portal vein thrombosis induced by disruption of portal vein inflow and stagnant blood flow because of mechanical effects from compression.^9^

Finally, the polycystic liver can become so large that it will cause compression of the inferior caval vein (ICV), resulting in ICVS. Oedema of both lower extremities can be the presenting symptom in case of ICVS.^10^

### 1.2 Incidence of portal hypertension in PLD

There is limited data on incidence of portal hypertension in PLD and these few reports are restricted to small cohorts which are heterogeneous or highly selected. In a retrospective study of 125 ADPLD patients, 6% developed portal hypertension during follow‐up.^11^ A single‐centre retrospective study from the United Kingdom found that of 47 PLD patients listed for liver transplantation, 40% had portal hypertension.^12^

The largest cause of portal hypertension in PLD patients is HVOO. The incidence has been investigated in a recent cohort study of preoperative imaging for 45 PLD patients undergoing liver resections. The study demonstrated that, based on a semiquantitative score, 78% of PLD‐patients had moderate stenosis while 22% had severe hepatic vein stenosis. Venous collaterals were present in the majority of patients.^9^ In addition to obstruction at the level of hepatic veins, the non‐cystic liver parenchyma of these patients may show so‐called ‘HVOO lesions’ at the level of the sinusoidal endothelium or terminal hepatic vein. These lesions (sinusoidal distension, congestion, peliosis and regenerative nodular hyperplasia) are seen in non‐cystic parenchyma in 92% of PLD patients and are also encountered in other diseases with HVOO.^9^, ^13^

In addition, the cohort study documented that liver biopsy findings were compatible with abnormal portal spaces in 67% of patients and portal vein dilation was present in 7% of patients.^9^ However, the incidence of clinically relevant PVO and ICVS is probably rare as the literature is limited to a few case reports.^10^, ^14^, ^15^, ^16^, ^17^ Interestingly, both presentations may be caused by a single, strategically located (very) large cyst.^10^, ^18^

### 1.3 Clinical symptoms of portal hypertension in PLD

The most common clinical symptom of portal hypertension in PLD is ascites. The accumulation of fluid within the peritoneal cavity further increases intra‐abdominal pressure leading to pressure‐related symptoms such as dyspnoea, abdominal distension, abdominal pain, increased weight and decreased quality of life of the polycystic patient.^15^, ^19^ As these overlap with symptoms caused by hepatomegaly in PLD, it can be challenging to discriminate between liver growth and ascites.^19^, ^20^

Cross‐sectional data show that 5% of ADPLD patients developed ascites during follow‐up.^11^ In a retrospective study of 461 ADPKD patients from South Korea, prevalence of ascites on imaging was 16.6% for the whole group. Importantly, presence of ascites was strongly correlated with liver volume, and more than half of severely affected PLD patients (hTLV ≥3200) were affected.^3^ In another study with PLD patients with portal hypertension that were listed for liver transplantation, nearly 58% had ascites.^12^

Besides liver volume, another important risk factor for the occurrence of ascites is abdominal surgery such as liver resections, laparoscopic fenestration or nephrectomy.^15^, ^21^, ^22^, ^23^, ^24^ For most patients, post‐operative ascites is transient and usually responds to medical management with diuretics, low salt diet and repeat paracenteses.^14^ After liver resection, 42% of patients had post‐operative ascites.^25^ Persistent and massive ascites was seen in 18%.^25^ In another study, refractory ascites after liver resection occurred in 9%.^14^ After laparoscopic fenestration, transient ascites occurred in 46% in one study, but was absent in other cohorts.^26^, ^27^, ^28^ We were unable to find data on refractory ascites after laparoscopic fenestration.

Finally, cyst rupture, a very rare complication of PLD, can also be the cause of transient ascites and is often accompanied by severe abdominal pain.^1^

Case reports have highlighted HVOO and refractory ascites as a complication of nephrectomy in ADPKD patients.^15^, ^21^, ^22^ In this respect, it is relevant to weigh the risks and benefits of nephrectomy, as the merits of nephrectomy and patient selection are uncertain.^29^ A 2015 guideline suggests that polycystic kidneys should not be routinely removed prior to transplantation, as it is associated with significant morbidity and mortality.^30^ Pretransplant nephrectomy is reserved for patients with a history of severe or recurrent cyst infections or bleeding, symptomatic nephrolithiasis, intractable pain and space restriction prior to transplantation.^30^ Post‐transplant unilateral nephrectomy appears to have fewer complications, but is also not without significant risks.^31^

It is not to be expected that etiology of polycystic disease forms an important risk factor. Unpublished data from our centre show that prevalence of ascites was evenly distributed among ADPKD (4%) en ADPLD patients (5%). In another cohort study there were no differences in ascites prevalence between patients listed for liver transplantation or combined liver‐kidney transplantation.^12^

The presence of varices and variceal haemorrhage is rare in PLD. In a retrospective study of 125 ADPLD patients, two (2%) patients had varices during follow‐up.^11^ The prevalence of varices was also 2% in patients listed for liver transplantation.^12^ Variceal haemorrhage has been described in only six cases.^32^, ^33^, ^34^, ^35^, ^36^, ^37^ For management of varices, we refer to the relevant guidelines.^38^, ^39^

## 2 DIAGNOSIS OF PORTAL HYPERTENSION IN PLD

### 2.1 Imaging

**Figure 3:**
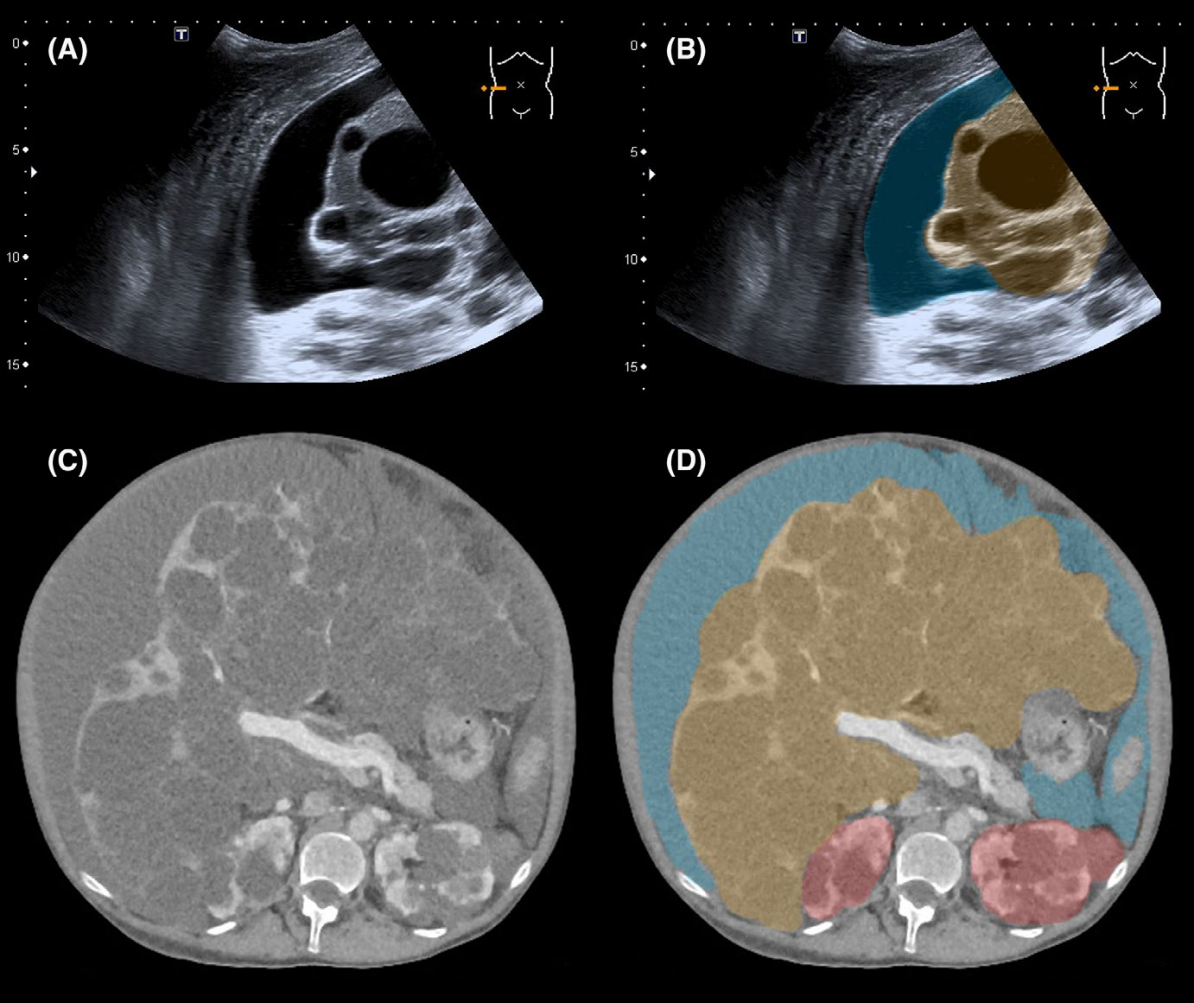
Imaging with ultrasound and transversal computed tomography (CT) of ascites in a polycystic liver disease patient with hepatic venous outflow obstruction. Ascites is coloured blue, polycystic liver tissue is coloured orange, polycystic kidney tissue is coloured red

Both hepatic cysts and ascites can be clearly distinguished with ultrasound (US), computed tomography (CT) and magnetic resonance imaging (MRI) (Figure 3).^40^, ^41^ In PLD‐patients presenting with increased abdominal swelling, ultrasound can be used to promptly distinguish between liver growth and accumulation of ascites. Splenomegaly can also be assessed with all three imaging modalities.^12^ Contrasted multiphasic CT or MRI can be used to show compression of the portal vein, hepatic veins and the ICV, while Doppler ultrasound is able to measure flow.^42^

### 2.2 Paracentesis

The role of paracentesis as a diagnostic tool is debated. Some suggest that initial workup in polycystic patients should include routine analysis of ascitic fluid to rule out infections.^42^ The discriminatory capacity beyond diagnosis of infectious processes is questionable. Ascites because of PLD can be both transudative^42^, ^43^, ^44^, ^45^ and exudative.^12^, ^18^, ^44^ Transudates result from increased fluid pressures in the plasma. Exudates can occur because of high permeability to proteins of the dilated sinusoidal walls in HVOO.^44^ As paracentesis does not distinguish between types of vascular obstruction, its merit only lies in the diagnosis of infected ascites.

### 2.3 HVPG

Even though HVPG is mandatory according to the definition of (clinically significant) portal hypertension, it is not universally performed in a standard fashion when evaluating cirrhotic patients.^46^ Furthermore, measurement of HVPG can be particularly technically challenging in patients with PLD because of the distorted anatomy.^12^ With lack of reporting of HVPG measurements in the literature, its use for PLD patients requires further clarification and validation in future studies.

## 3 MANAGEMENT OF PORTAL HYPERTENSION IN PLD

Reduction in portal pressure is achieved by decrease in portal flow by splanchnic vasodilation. Beta blockers are the cornerstone of treatment of portal hypertension, however, there is no literature that documents the benefit of propranolol or carvedilol in context of PLD.^38^ Somatostatin analogues (SSA), such as octreotide or lanreotide, also reduce hepatic blood flow and portal pressure and are often used in PLD patients because of the ability to reduce liver volume.^1^ However, the reported effects on lowering portal pressure have been variable with a majority of studies in general cirrhotic patients reporting little to no effect.^47^ Octreotide is only recommended in case of variceal bleeding.^38^

This review on the management of portal hypertension in PLD focusses on (1) the management of ascites by SSA, diuretics and paracentesis, (2) the percutaneous or surgical reduction in liver volume (3) the restoration of flow in the liver vasculature by stents and shunts. Finally, liver transplantation will be discussed. It is important to consider liver transplantation assessment in an early stage, and should be performed in parallel to the management options described below.

### 3.1 Management of ascites

While the effect of SSA on abdominal complaints and liver volume has been studied in PLD, little research has been done to study the merits of therapy for ascites. One study described two cases of PLD‐associated ascites that were successfully treated with SSA. In both patients, this resulted in a dramatic clinical improvement, disappearance of ascites and a decrease of liver volume, without the need for interventional treatment.^48^ As side effects of SSA are usually mild and diminish over time, they can be used as a valid alternative to more invasive procedures in PLD.^1^ Some authors have proposed treatment with SSA in combination with ranitidine after fenestration surgery to minimize development of ascites through the surface of the exposed cyst remnants, but more research is needed to support this strategy.^49^

Ursodeoxycholic acid (UDCA) has also been proposed as medical therapy for PLD, as it inhibits cholangiocyte proliferation in vitro and in murine models.^50^, ^51^ However, a phase‐2 randomized controlled trial showed no benefit on growth of total liver volume.^52^ We were unable to find any data on the effect of UDCA on ascites or portal hypertension in PLD.

As with ascites in decompensated cirrhotic patients, diuretics are used to decrease production of ascites. Although sodium restriction has not been studied in PLD‐patients, it seems reasonable to advocate this practice in line with guidelines for other causes of ascites.^38^, ^53^ Spironolactone and furosemide are indicated in cirrhosis as the Renin‐Angiotensin‐Aldosteron‐System (RAAS) is markedly upregulated and patients develop a hyperdynamic circulatory syndrome.^54^, ^55^ However, this is not necessarily the case for PLD‐related ascites. We could not identify studies that investigated RAAS in PLD, but the mechanisms of HVOO in PLD are likely to be comparable to BCS. One study found that haemodynamics are markedly different between BCS patients and matched decompensated cirrhosis patients. Patients with BCS had normal cardiopulmonary haemodynamics, and most of them did not exhibit systemic vasodilation, but nonetheless had a marked activation of neurohumoral vasoactive systems (such as RAAS).^54^ In addition, patients with ADPKD are predisposed to early onset hypertension, which has been attributed, among other factors, to activation of RAAS by the enlargement of renal cysts.^56^ Since RAAS upregulation and the resulting hyperaldosteronism also seem to play a pivotal role in hepatic vein obstructions, spironolactone should typically be the first‐line diuretic. Chlorothiazide or furosemide can be added, which can provide synergy and avoid hyperkalaemia.^38^

Vaptans are selective antagonists of the vasopressin 2 (V2) receptors in the principal cells of the collecting ducts that enhance solute‐free water excretion and thus raise serum sodium levels.^53^ For example, tolvaptan has been shown to have a survival‐benefit compared to control.^57^ Additionally, a phase 2 trial has shown that tolvaptan significantly reduced the body weight and abdominal circumference compared to placebo in patients with liver cirrhosis‐associated ascites.^58^ Another randomized trial showed that a combination of conventional natriuretic drugs and tolvaptan was superior to conventional therapy alone in cirrhosis‐associated ascites.^59^

Two recent case reports have suggested that tolvaptan may also reduce liver volume.^60^, ^61^ The potential effect was corroborated by an in vitro study that showed involvement of vasopressin in liver cyst growth.^62^ The effect of tolvaptan on liver volume in PLD is currently being investigated in larger cohorts. Tolvaptan is also effectively used in ADPKD patients with rapidly progressive disease to slow deterioration of renal function.^63^ So in theory, ADPKD patients with PLD‐related portal hypertension could benefit particularly from tolvaptan treatment.

It is important to note that the safety of vaptans for cirrhotic patients has only been established for short‐term treatments lasting from one week to one month.^38^ Thus, at present, the use of vaptans for portal hypertension is limited to controlled clinical studies.^38^ As liver function is preserved in PLD patients, we hypothesize that the risks of vaptans is lower in this group and its use for PLD‐related portal hypertension needs further investigation. However, the high cost of tolvaptan, which ranges between € 15 000 and € 30 000 per year, is a major barrier for widespread use.^64^, ^65^, ^66^

Lastly, large volume paracentesis (with or without albumin replacement) under radiological guidance should be used to achieve symptomatic relief, reduce fluid burden and alleviate abdominal distension.^15^, ^45^ The presence of spontaneous bacterial peritonitis, although infrequent, should be considered.^38^ We were unable to find data on the prevalence of peritonitis in PLD patients.

### 3.2 Reduction in liver volume

Liver‐volume reducing therapy is the mainstay of treatment for PLD. Current guidelines advocate the use of SSA for this purpose.^1^ Multiple studies have shown that SSA reduce liver volume by 3%‐8% compared to an increase in liver volume in the control group of 1 to 8%.^1^ Besides medical treatment with SSA, several percutaneous (sclerotherapy and embolization) and surgical (fenestration and resection) interventions are used.^1^ In specific cases, these interventions can also be used to treat strategically located cysts or reduce mass effect. Subsequent improvement of hepatic blood flow reduces portal hypertension.

Percutaneous aspiration sclerotherapy is a valid strategy for treatment of large symptomatic hepatic cysts. A pigtail catheter is positioned in the cyst cavity to evacuate the fluid. Next, a sclerosing agent (eg ethanol, tetracycline, polidocanol) is injected, which damages the inner epithelial lining resulting in regression of the cyst.^67^ A recent systematic review found that aspiration sclerotherapy reduces proportional cyst volume by 76%‐100%.^68^ Aspiration sclerotherapy comes with complications such as pain, ethanol intoxication, cyst bleeding and rarely cyst infections.^68^ Because of its minimally invasive nature and potency to achieve cyst volume reduction, aspiration sclerotherapy can be used to treat strategically located cysts that are the cause of portal hypertension. In a case report, in one patient with ascites and massive oedema of the lower extremities, three strategically located cysts were aspirated to relieve caval pressure. Additionally, ascites was drained, diuretics and somatostatin analogue were started and the patient recovered,^10^ highlighting that a combination of conventional and interventional treatment is often necessary. In a second patient with portal hypertension, a large gastro‐renal shunt and liver dysfunction, a total of 13 aspiration sclerotherapy procedures were used to reduce liver volume. Afterwards, balloon‐occluded retrograde transvenous obliteration of the shunt and partial splenic embolization were performed to increase portal blood flow, which resulted in restoration of liver function.^69^

A novel intervention to reduce liver volume is transcatheter arterial embolization (TAE). Therapy comprises placement of microcoils in hepatic artery branches and may be an option for treatment of patients in poor functional status with symptomatic PLD.^70^ TAE was first described in 2004. In this case report, two TAE procedures were performed in a patient with massive ascites who needed therapeutic paracentesis every two weeks. The need for any paracenteses subsided after the second intervention. Liver volume was reduced by 54% after 2 years of follow‐up.^71^ TAE may be an alternative to liver resection, however, only retrospective studies have been performed and very few centres are experienced with this procedure.

An alternative to the percutaneous approach is laparoscopic fenestration, sometimes also called deroofing. It combines cyst fluid aspiration and surgical excision of extra‐hepatic cyst wall in a single laparoscopic procedure. A recent systematic review reported the effectiveness for solitary cysts and PLD patients. The recurrence rate (34%) and complication rate (29%) in PLD patients was high. An estimated 7% of PLD‐patients undergoing laparoscopic fenestration suffered major complications.^23^ Laparoscopic fenestration is also a risk factor for ascites. Aspiration sclerotherapy and laparoscopic fenestration have never been compared head‐to‐head in a formal clinical trial. Indications, techniques and follow‐up vary between centres and studies, so it is difficult to compare the volume‐reducing ability of the procedures. In our centre, there is a preference for aspiration sclerotherapy as it is less invasive and carries a lower complication rate.^72^ Laparoscopic fenestration is used after second recurrence of cyst growth or if more than two large cysts need to be treated for a relevant effect. Specialized hepatologists, surgeons and interventional radiologists should make a comparative assessment on gains and risks for the individual patient. The effectiveness of laparoscopic fenestration for treatment‐resistant ascites has not been described in the literature.

Beyond laparoscopic fenestration, there is more extensive surgery that can be applied to PLD. Liver resection consists of resection of multiple liver segments and is often combined with cyst fenestration of the remnant liver. Liver resection is the only therapy that guarantees a large reduction in liver volume.^24^ However, major morbidity occurs in 21% of patients and operative mortality is 3%. Importantly, liver resection was the cause of treatment‐resistant HVOO that required vascular intervention in 5% of patients.^24^ A few published cases (one with PVO and three with ICVS) underline the potential of (extended) liver resection for ascites in PLD.^10^, ^15^

Two classifications that guide decision‐making for surgical therapy in PLD have been proposed. The Gigot classification (type I, II & III)^73^ and Mayo classification (type A, B, C & D)^74^ both categorize patients based on number, size and location of hepatic cysts. Observation or medical therapy is advised for type A patients. Cyst fenestration is recommended for type B or type I/II patients. Combined partial hepatectomy and cyst fenestration is performed in type C or type III patients. Liver transplantation can be considered for type D patients. However, the classifications have not been tailored to PLD patients suffering from portal hypertension or ascites because of liver cysts.

### 3.3 Restoration of flow

In a number of PLD patients, conventional therapy for portal hypertension is insufficient, refractory ascites has ensued, and the limits of volume‐reducing therapy have been reached and preclude repeat operative intervention. Also, imaging of the portal and hepatic veins or ICV may have identified a focal point of compression/stenosis leading to venous obstruction. These clinical settings should prompt the consideration of intravascular stenting. In addition, the use of surgical portocaval or percutaneous peritoneovenous shunt creation is discussed.

In case of ICVS and/or HVOO, patients may be treated with percutaneous venous stenting to relieve venous obstruction. One retrospective study found seven patients that were treated with ICV stenting and two patients had concomitant right hepatic vein stenting. All patients presented with refractory ascites. Five (71%) of patients had maintained clinical improvement after a mean follow‐up of 12 months. In the two non‐responders, surgical peritoneovenous shunt creation was necessary.^14^

Only two case reports describe the use of hepatic vein stenting without ICV stenting. Both patients presented with intractable ascites because of HVOO and were treated with self‐expanding metal stents. The first patient had normal caliber left and right hepatic veins and stenosis of the middle hepatic vein because of multiple cysts, which was treated with stent placement. The second patient had absence of contrast enhancement in the left and middle hepatic veins, and only minor flow in the right hepatic vein. In this case, only canalization of the right hepatic vein could be established to facilitate stent placement. In both patients the pressure gradient restored to normal values after stent placement and patients showed a swift recovery with disappearance of ascites.^45^, ^75^

Literature on stent placement for PVO is limited to one report. This describes a PLD patient with recurrent variceal haemorrhage because of portal vein stricture that was treated with balloon dilation and placement of a 14 mm Wallstent in the portal vein. In addition, gastric and oesophageal varices were embolized.^32^

These results suggest that venous stent placement is safe and effective in select patients and should be considered as a possible intervention in the treatment of medically intractable ascites.

Transjugular intrahepatic portosystemic shunts (TIPS) decompresses the portal system by shunting the portal system into a hepatic vein. The clinical effects of TIPS for decompensated cirrhotic patients have been confirmed in prospective randomized controlled trials and meta‐analyses have concluded that TIPS controls ascites better than large‐volume paracentesis.^38^ However, presence of PLD has been described as an contra‐indication for the use of TIPS for portal hypertension as the creation of an intrahepatic tract may involve traversal of a cyst.^76^ The potential consequences of cyst puncture may include (severe) haemorrhage, and instability if the stent bridges a cyst cavity rather than parenchyma. In addition, the effect of contact between the stent and cyst fluid, or a blood‐filled cyst cavity, on shunt patency is unknown.^77^, ^78^

In contrast, some authors have questioned this contra‐indication.^76^ The use of TIPS for PLD is limited to a few case reports, and has been described as successful.^76^, ^78^, ^79^, ^80^, ^81^, ^82^ However, complications such as intracystic haemorrhage, spontaneous bacterial peritonitis and encephalopathy have also been reported.^82^ The use of a hybrid 2D/3D imaging instruments or intravascular US guidance can be used to increase safety of TIPS placement in the setting of PLD.^78^, ^80^, ^82^ Thus, TIPS placement in context of PLD should be approached with caution. There are risks and technical challenges, and evidence of feasibility is based on a limited number of case reports. When TIPS is considered it should be preferably performed in expert centres with experienced teams and advanced imaging‐guidance systems.

The more widespread use of liver transplantation and TIPS has superseded the use of surgical portocaval shunts, which consist of surgical stent graft placement between the portal and caval venous systems.^83^ The decline is explained by the high mortality rate of 20%‐50%,^84^ which may be even higher in the present because of a world‐wide decline of experience with these procedures. The placement of a surgical mesocaval and portocaval shunt has been reported for PLD, with no procedure‐related mortality and disappearance of ascites. However, no long‐term follow‐up was described.^15^, ^44^ Mesocaval shunts can also be placed percutaneously by an interventional radiologist.^84^ But to our knowledge, this procedure has not been used for PLD patients as of yet.

The use of peritoneovenous shunts for decompensated cirrhotic patients with refractory ascites was popularized in the 1970s, but has been largely abandoned in recent years because of poor long‐term results and excessive complications.^53^ According to the American Association for the Study of Liver Diseases guideline, peritoneovenous shunting is reserved for diuretic‐resistant decompensated cirrhosis patients who are not candidates for transplant or TIPS.^53^ One case report, published in 1986, describes a patient with PLD and renal cysts. Symptoms and ascites resolved after peritoneovenous shunt placement and renal function had also improved at 14 months follow‐up.^85^

In our opinion, performing surgical shunts is only indicated in severely affected PLD patients that are not eligible for liver transplantation. Potential benefits and risk should be carefully considered. Surgery should be performed in centres with extensive expertise in vascular surgery and surgical graft placement.

### 3.4 Liver transplantation in PLD

Liver transplantation is the only curative treatment option for PLD. As liver function is preserved in almost all patients, exception criteria have been formulated: transplantation is indicated in case of massive hepatomegaly and poor quality of life, in combination with a complication that is likely to resolve after liver transplantation. Specified complications include severe malnutrition, cachexia, biliary obstruction, cholestasis, recurrent cyst infections, and importantly: refractory ascites, portal hypertension, variceal bleeding or HVOO.^86^ Data from the European Liver Transplantation Registry show a high five‐year graft survival (88%) and patient survival rate (92%) for transplanted PLD patients.^87^

Liver transplantation should be considered in patients with refractory ascites that is not amenable to treatment with conventional measures, liver‐volume reducing therapy and vascular stenting or shunting. We advise to refer patients promptly for assessment for liver transplantation to prevent any delays. Combined liver‐kidney transplantation in ADPKD patients with severe renal impairment should also be considered.^1^

## 4 CONCLUSION

The evidence base supporting treatment options for portal hypertension and ascites in PLD is limited, and primarily consists of case reports. Thus, all recommendations have an evidence level of D according to the GRADE criteria, and should be read as an expert opinion.^88^ The use of liver transplantation in PLD has been studied in large cohort studies, resulting in moderate quality of evidence (grade B). It is important to consider liver transplantation assessment in parallel to alternative treatment options for ascites and portal hypertension.

**Figure 4:**
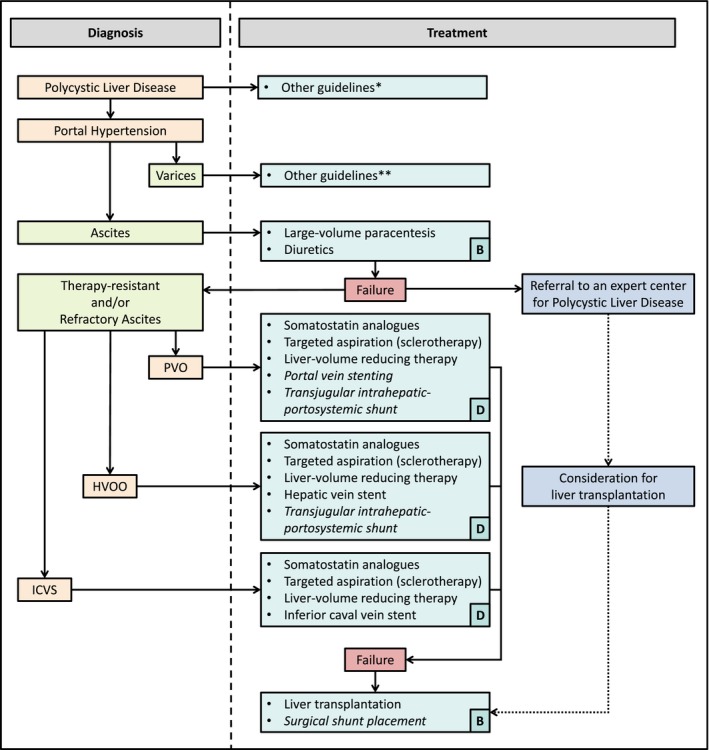
Treatment flowchart. Diagnoses are marked in orange, symptoms in green, treatment options in blue, failure of therapy in red. Treatment options are italicized as a caution when caveats apply. Assessments for liver transplantation should be performed in parallel to other therapies. Level of evidence is shown in the lower right corner according to the GRADE criteria (A = high, B = moderate, C = low, D = very low). Abbreviations: HVOO, hepatic venous outflow obstruction; ICVS, inferior caval vein syndrome; PVO, portal vein obstruction. Asterisks: *See reference[1]. **See references [38, 39]

We propose the following algorithm for treatment of portal hypertension in PLD (Figure 4). When ascites is present, first treatment should consist of conventional therapy with diuretics. Large‐volume paracentesis can be performed to provide symptomatic relief. When this is insufficient, patients are suffering from therapy‐resistant and/or refractory ascites. In this case, further diagnostics are warranted and could consist of US, CT or MRI imaging. Depending on the presence of PVO, HVOO, ICVS or a combination, treatment should be tailored to the individual patient. When somatostatin analogues, percutaneous interventions, surgery, venous stenting and TIPS are not possible or not efficacious, liver transplantation should be performed, which is also curative therapy. If patients are not eligible for liver transplantation, surgical shunt placement might be an alternative, but has a serious morbidity and mortality rate, especially in less experienced hands.

## CONFLICT OF INTEREST

The authors do not have any disclosures to report.
